# Iron—sepsis associations in population health revealed by epidemiology

**DOI:** 10.1016/j.ebiom.2025.105927

**Published:** 2025-09-15

**Authors:** Randi Marie Mohus, Lise T. Gustad, Jan Kristian Damås, Hal Drakesmith

**Affiliations:** a Central Norway Sepsis Research Centre, Norwegian University of Science and Technology, Trondheim, Norway; b Clinic of Anesthesia and Intensive Care, St. Olavs Hospital, Trondheim, Norway; c Department for Circulation and Imaging, Norwegian University of Science and Technology, Trondheim, Norway; d Faculty of Nursing and Health Sciences, Nord University, Levanger, Norway; e Department of Medicine and Rehabilitation, Nord-Trøndelag Hospital Trust, Levanger, Norway; f Clinic of Medicine, Department of Infectious Diseases, St. Olavs Hospital, Trondheim, Norway; g Department of Molecular and Clinical Medicine, Norwegian University of Science and Technology, Trondheim, Norway; h MRC Translational Immune Discovery Unit, MRC Weatherall Institute of Molecular Medicine, University of Oxford, John Radcliffe Hospital, Oxford, OX3 9DS, UK

**Keywords:** Iron status, Iron deficiency, Iron overload, Sepsis, COVID-19, Bloodstream infections, Epidemiology

## Abstract

Sepsis is a leading cause of death and disability worldwide, so identifying preventable risk factors is important. Iron is essential for immune function and microbial growth, and iron status varies substantially between individuals, across demographics, and is therapeutically modifiable. Here, we review the current understanding of iron status and associated risks of bloodstream infection, sepsis and severe COVID-19 highlighting relevant population-based studies and Mendelian randomisation studies. Both low and high iron status are associated with increased risk of sepsis. Low iron status is associated with sepsis, bloodstream infections and pneumonia. High iron status and mutations affecting hepcidin regulation are linked to increased risk of bloodstream infections, sepsis and COVID-19. Both iron status pathologies and sepsis are global health issues, and the epidemiological studies described indicate they may be linked. More population-scale investigations on iron status, infection and immunity, especially in areas of high iron deficiency and infectious burden are warranted.

## Introduction

Despite advances in care and treatment, sepsis remains a global health priority due to its high morbidity and mortality.^1^^,^^2^ Invasive bacterial infections and systemic viral infections such as COVID-19 may both evolve to and share common manifestations with sepsis.^3^, ^4^, ^5^ The role of iron pathologies, which are among the most common micronutrient disorders,^6^, ^7^, ^8^, ^9^ in increasing the risk of infections and sepsis is still debated. There are indications that both low and high iron status might affect an individual's risk of acquiring an infection,^10^, ^11^, ^12^, ^13^, ^14^, ^15^ as well as the course and severity of the infection.^11^^,^^12^^,^^16^, ^17^, ^18^, ^19^, ^20^, ^21^, ^22^ There is a worrisome lack of population studies assessing iron status and risk of sepsis in regions where the prevalence of iron deficiency and infection risk are highest.^7^^,^^9^ An increasing body of evidence supports that iron status disturbances play an important role in the pathophysiology of sepsis,^20^^,^^23^ bacteraemia,^24^ and COVID-19.^17^ With COVID-19, a large body of literature identified iron metabolism dysregulation as a hallmark of the pathogenesis of severe cases,^17^^,^^19^^,^^22^ and the likelihood of developing ‘Long Covid’.^16^^,^^25^ A recent population study assessed carriers with mutations in the *HFE*, a gene involved in hepcidin regulation, identified an increased risk of infections even in carriers with normal iron status.^11^ Impaired hepcidin regulation could affect iron homoeostasis leading to higher susceptibility to infections.^26^ Therefore, preventing disturbances in iron status and modifications of iron status during severe infections,^27^^,^^28^ might improve prognosis of severe infections.^14^^,^^29^ In this review, we aim to synthesise epidemiological findings, assessing population-based and Mendelian randomisation (MR) studies, to evaluate how iron status influences risk of future hospitalisations with bloodstream infection (BSI), sepsis and COVID-19 in adult populations.

### Iron status and important immune functions

Adequate iron levels must meet the needs of different tissues, excess iron causes cellular damage, therefore the maintenance of iron homoeostasis is essential. Iron influences nearly every biochemical processes (e.g., cellular respiration, synthesis of oxygen-carrying molecules, gene regulation, cell growth regulation, cell differentiation, co-factor in enzymatic reactions), and is crucial in red blood cell formation, immunity, foetal development, and physical and mental well-being.^30^^,^^31^ In healthy humans, the plasma iron concentration is stable and bound to transferrin (normally 20–40% saturated) which serves as a buffering system to avoid free iron. However, only 0.1% of the body's iron content is in plasma, most body iron is bound to haem (approximately 70%), and in the storage molecule ferritin. Ferritin encloses iron within a molecular core, protecting it, maintaining it in solution and ensuring biological availability.^32^ Iron is stored in hepatocytes and in splenic and hepatic macrophages.

Systemic iron trafficking is orchestrated by hepcidin, which reduces iron absorption and enhances cellular iron storage. Reduced levels of hepcidin will normalise iron absorption and iron release from storage. Inflammation increases hepcidin leading to hypoferraemia which develops within hours of a systemic infection, thereby reducing extracellular iron available for invading extracellular pathogens.^33^^,^^34^ This hypoferremia also provides an increased capacity to bind iron that has been released by tissue destruction at sites of infections.^34^ For intracellular pathogens, other iron-oriented strategies come into play, such as ferroportin mediated iron export from macrophages, shown to reduce the microbial load of some bacteria thriving in macrophages.^28^^,^^31^^,^^33^ Persistent iron restriction can eventually reduce iron availability to host cells, inhibit erythropoiesis and lead to “anaemia of inflammation”.^35^ The optimum level of host iron status and iron distribution might be different from pathogen to pathogen,^36^^,^^37^ and dependent on other medical conditions.^35^

Studies have shown that iron deficiency (ID) and iron overload affect various aspects of innate and adaptive immune functions such as: 1) ID is associated with reduced function and decreased myeloperoxidase activity in neutrophils, whereas iron overload decreases the antibody-mediated and mitogen-stimulated phagocytosis by monocytes and macrophages, 2) ID is associated with reduced production of macrophages’ migration inhibition factor, 3) ID leads to decreased and defective T-cells and reduced lymphoproliferation, and impaired IL-2 production by lymphocytes. On the other hand, iron overload alters numbers and subset of T-cells with potentially increased CD8/CD4 ratio, and may affect immunoglobulin secretion, 4) ID decreases cytokine production and natural killer cell activity, 5) reduced production of TNF-alpha by monocytes is seen in haemochromatosis patients, 6) reduced bactericidal activity in macrophages due to iron being a critical component of the “oxidative burst”, and 7) iron overload is associated with suppressed functions of the complement system.^31^^,^^38^, ^39^, ^40^, ^41^, ^42^ The knowledge of potential effects on immune functions from longstanding ID remains incomplete.^24^ For malaria, there is evidence that ID in children and in pregnant women protects against infection,^43^^,^^44^ but persisting ID in childhood has other detrimental health effects (e.g., failure to thrive, inhibition of cognitive development, and immune impairment).^9^

Excess iron can be detrimental by promoting pathogen growth.^45^ Iron overload compromises the bactericidal capacity of phagocytic cells and decreases chemotactic responses. Invading bacteria have different mechanisms to acquire iron including direct uptake of iron; haem or haem-containing moieties; chelation of iron using siderophores; and capture of transferrin or lactoferrin via bacterially encoded receptors. Some intracellular bacteria may thrive due to the hepcidin induced shift of iron into macrophages.^33^^,^^46^^,^^47^ The study by Barber and Elde demonstrated bacterial mechanisms to get iron from transferrin. Importantly, they showed that the battle for iron has driven transferrin evolution through positive genetic selection in the host–pathogen interface.^48^ Excess “free iron” in the cytoplasm, caused by haemolysis and erythrophagocytosis, can make iron more available for pathogen acquisition. Further, iron excess may aggravate inflammation due to iron's capacity to generate toxic free radicals, inducing cell death which has been linked to multi-organ failure and death.^27^^,^^29^

Nutritional iron supplementation has been associated with acute exacerbations of infections, particularly in children in malaria-endemic regions,^49^ but also confirmed in a meta-analysis of intravenous iron supplementation in hospitalised populations.^50^ In a study of patients with inflammatory bowel disease, increased risk of bacteraemia was identified in patients receiving oral iron supplements prior to hospitalisation.^51^ A pre-clinical study showed that iron deficient anaemic mice given iron supplements had higher bacterial loads and a worse outcome after injection with Salmonella Typhimurium.^37^ The dilemma concerning iron supplementation with the potential risk of infections versus the health effects of ID in Sub-Saharan children was reviewed by Jonker et al.^52^ Another concern is that oral iron supplementation and fortification may prove less effective in areas of high infectious burden where elevated hepcidin levels could limit iron absorption.^53^

### Iron status and severe infections

Assessing iron status in the population is challenging as there is no single, reliable iron biomarker. Several iron markers are in use to assess iron status (e.g., serum iron, transferrin saturation (TSAT), total iron binding capacity (TIBC), ferritin, transferrin, reticulocyte count, hepcidin, haemoglobin, and soluble transferrin receptor) (Table 1). Each marker has its strengths and weaknesses depending on physiological conditions and infrastructure at the blood sampling site.^6^^,^^54^ Another challenge with iron measurements is the distinction between absolute ID (i.e., a severe reduction or absence of iron stores) or a functional ID (i.e., insufficient iron availability despite adequate iron stores).^55^ The general risk of infections appears to follow a U-shaped curve, where both low iron status (i.e., low serum iron, low TSAT, high TIBC, and/or low ferritin indicating ID) and high iron status (i.e., high serum iron, high TSAT, low TIBC, and/or high ferritin) are linked to increased risk of infection.^10^^,^^11^^,^^14^^,^^18^ Measurements of ferritin could be difficult to interpret during infection or inflammation as this is an acute phase reactant.^54^ Emerging dynamic biomarkers, like hepcidin and Ferritin/CRP ratio, demonstrate promising usability to diagnose ID in presence of inflammation and to enhance iron treatment.^56^

**Table 1:** Iron status markers and their association with iron deficiency and high iron status.

Iron marker	Indicating iron deficiency	Indicating high iron status
Serum iron	Low	High
Transferrin saturation (TSAT)	Low	High
Transferrin	High	Low
Ferritin	Low	High^a^
Reticulocyte count	Low	b
Haemoglobin	Low	b
Hepcidin	Low	High^c^
Total iron binding capacity (TIBC)	High	Low
Soluble transferrin receptor	High	Low

a Ferritin levels increase as an acute phase marker.

b Not necessarily affected by high iron status.

c Levels of hepcidin in haemochromatosis are dependent on the mutation.

Critical illness triggers an inflammatory response which may result in profound changes to iron metabolism.^21^^,^^57^ In sepsis, there is an enduring and simultaneous pro- and anti-inflammatory state driven by a dysfunctional innate and a suppressed adaptive immune response eventually leading to the “cytokine storm”.^58^ In the early stages of sepsis the release of pro-inflammatory cytokines leads to iron sequestration. Emerging evidence suggests that high intracellular iron might activate inflammatory signalling pathways leading to cell death and tissue damage,^29^ eventually through ferroptosis, a process of iron-dependent programmed cell-death.^27^ Studies assessing iron status at intensive care unit (ICU) admission showed that iron status deviations were associated with decreased short- and long-term survival for sepsis patients.^20^^,^^59^^,^^60^ Iron status in sepsis has been reviewed by Liu et al., during sepsis there is altered iron uptake, transport and export.^29^ Septic patients face a catabolic state with decreased iron consumption, but increased iron release from destroyed tissues. This hyperferremia could mean that the body's chaperoning systems are overwhelmed, further exacerbating the ongoing tissue damage and the development of multi-organ failure.^27^^,^^29^

Accumulating evidence supports that iron metabolism dysregulation is a characteristic of severe COVID-19.^17^ Studies relate hyperferremia,^18^ hyperferritinemia,^17^^,^^22^ but also low iron status,^21^^,^^61^^,^^62^ to a more severe course of COVID-19. A meta-analysis found evidence of lower serum iron and TIBC levels, but higher ferritin levels in deceased COVID-19 cases.^17^ A retrospective investigation of iron homoeostasis at hospitalisation found that a ferritin/transferrin ratio >10 predicted higher risk of ICU-admission and being mechanically ventilated.^22^ While restricting iron to invading pathogens may be protective in the short term, it could become harmful in the long term. The reduced iron availability in the convalescent period of critical illness is associated with functional ID due to high levels of hepcidin.^63^ This has been linked to cognitive, neuromuscular, and cardiopulmonary dysfunctions, anaemia, and poor quality of life after critical illness.^57^ Sonnweber et al. found that after COVID-19 infection there are persisting alterations of iron homoeostasis (absolute ID or functional ID based on TSAT and ferritin) persisting for at least two months and associated with impaired physical performance and non-resolving lung pathologies.^25^ In a study of post-acute sequelae (PASC) (i.e., “Long covid”) by Hanson et al., prolonged inflammation and disrupted iron homoeostasis persisting for more than two weeks post-COVID-19 was associated with patients reporting PASC several months later.^16^

The variance in reported hypo- and hyperferremia of septic patients and severe COVID-19 patients could reflect the extensive changes to iron metabolism during an infectious threat, and how iron metabolism is orchestrated by the host and the pathogen. The timing of iron status measurement is of importance, and the relatively small numbers of included patients. Therefore, an overall investigation of the current available population-based epidemiological and population genetics research is needed, linking different types of evidence on altered iron status and risk of severe infections.

## Search strategy and selection criteria

We searched MEDLINE, Cochrane Register and Google scholar with combinations of search terms including (“iron” OR “iron deficiency” OR “iron overload” OR “iron metabolism disorders”) AND (“sepsis” OR “bacteremia” OR “COVID-19”). We also included search with (“iron” OR “iron deficiency” OR “iron overload”) AND (“population” OR “public health” OR “epidemiology” OR “mendelian randomis [z]ation”). In Google scholar we included full text searches for “iron status risk of sepsis”, “iron status risk of bloodstream infection”, “iron status risk of COVID-19” and “iron status mendelian randomis [z]ation study”. Databases were searched from inception until Jan 20, 2025. We included all population-based studies that investigated risk of severe infections related to iron status measured at enrolment, or MR studies that assessed the future risk of BSI, sepsis or COVID-19 related to iron status. Inclusion by the same criteria were also made by examining the references of the identified articles.

## Iron status as a risk factor for BSI and sepsis in population studies

Despite the obvious important role of iron in human health and diseases,^64^ very few population studies assessing iron status at the population level have been conducted.^6^ Population-based studies are epidemiological studies in which participants are sampled from a well-defined general population, rather than selected sub-groups, to ensure representativeness and minimise selection bias. They are important to raise knowledge about health issues in the general population.^65^ To date, two large population studies assessing iron status and risk of severe infections have been published; one Danish population study that included 142.188 adult participants followed for a median of eight years,^11^ and our study from 2018 investigating 61.852 participants from the Norwegian HUNT2 cohort with a median follow-up of 14.8 years.^10^ Study participants had blood sampling (including iron status measurements), physical examination and filled out questionnaires regarding health and lifestyle at inclusion in both studies. Iron status measurements in the Danish study included serum iron, TSAT and ferritin and levels of the iron indices were divided into five categories using percentiles; 0‒5th, 6th‒25th, 26th‒74th, 75th‒94th and 95th‒100th.^11^ Our study included serum iron, TSAT and TIBC, and categorisation was based on the distribution in the entire HUNT2 cohort and divided into seven groups; ≤2.5th percentile or ≥97.5th percentile, and the values in between were categorised into quintiles.^10^

Mottelson et al. included the covariates sex and age from the Danish Civil Registration System. Smoking status, cumulative smoking in pack-years, alcohol intake, and menopausal status were taken from the questionnaires, body mass index was from the physical examination and C-reactive protein (CRP) levels were from laboratory measurements at enrolment. Information on comorbidities was achieved from the Danish National Patient register at enrolment.^11^ Our study retrieved information on sex, age and date of death or emigration from the Norwegian Population Register, and derived information from the self-reported questionnaires in HUNT2 on cardiovascular disease, lung disease and diabetes. Chronic kidney disease was defined by estimated glomerular filtration rate <60 ml/min/1.73 m^2^, and body mass index was from the physical examination. We retrieved information on cancer diagnoses from the Cancer Registry of Norway for all patients with BSI before and during follow-up. We also retrieved information on rheumatic illnesses and inflammatory bowel disease from medical records of patients with BSI during follow-up.^10^

The two studies had BSI as outcome, the Danish study additionally studied risk of pneumonia and sepsis. Mottelson et al. obtained outcome data as admissions with an infectious main diagnosis from the Danish National Patient Register, information on BSI was obtained from the Danish Microbiology Database, and death with an infection as the underlying cause of death was retrieved from the Danish Civil System. In HUNT2 all participants were followed for BSI episodes identified at the three hospitals in this region of Norway and registered in the Mid Norway Sepsis Registry until end of follow-up, death or migration out of the region whichever occurred first. BSI mortality was defined as death occurring within 30 days after detection of a BSI. Detailed information is provided in the manuscripts and their Supplemental Materials.^10^^,^^11^

Adjusted Cox regression was used to estimate hazard ratios (HR). The categorisation of iron status measurements differs making a pooled estimate in a meta-analysis inappropriate. Both studies pointed to a U-shaped risk, i.e., a higher risk of infections associated with extreme low and high iron status, between most of the iron parameters analysed and infection risk outcomes. As shown in Table 2, low serum iron (≤7.1 μmol/L), high serum iron (≥22.5 μmol/L) and high TSAT (>39%) was associated with higher risk of BSI when compared to the middle category (26th─74th percentiles) of each measured iron indices in the Danish Study. The same pattern was found for increased risk of sepsis. Risk of pneumonia was associated with low and high serum iron, and low TSAT (<11%). There were no clear associations between ferritin levels and risk of severe infections. When investigating risk of death from any infectious disease, low serum iron showed an HR of 1.31 (CI 1.14–1.52), and the HR of low TSAT was 1.31 (CI 1.12–1.53).

**Table 2:** Association between iron status indices and the risk of BSI, sepsis and pneumonia in population studies.

	Mottelson et al. risk of BSI^11^^,^^a^	Mohus et al. risk of BSI^10^^,^^b^	Mottelson et al. risk of sepsis^11^^,^^a^	Mottelson et al. risk of pneumonia^11^^,^^a^
**Total population**	131.376 (iron) 131.326 (TSAT) 31.838 (ferritin)	61.852 (iron, TSAT, TIBC)	136.656 (iron) 136.599 (TSAT) 38.020 (ferritin)	136.656 (iron) 136.599 (TSAT) 38.020 (ferritin)
**Numbers of severe infections**	3162 (iron) 3161 (TSAT) 906 (ferritin)	1738 (iron, TSAT, TIBC)	3530 (iron) 3530 (TSAT) 1136 (ferritin)	8890 (iron) 8885 (TSAT) 3470 (ferritin)
Low Se iron HR (95% CI)	1.23 (1.05–1.44)	1.72 (1.34–2.21)	1.18 (1.01–1.38)	1.20 (1.10–1.32)
High Se iron HR (95% CI)	1.26 (1.08–1.46)	1.06 (0.72–1.57)	1.38 (1.20–1.58)	1.22 (1.11–1.34)
Low TSAT HR (95% CI)	1.15 (0.98–1.36)	1.48 (1.12–1.96)	1.12 (0.95–1.32)	1.20 (1.09–1.32)
High TSAT HR (95% CI)	1.24 (1.07–1.44)	0.84 (0.59–1.20)	1.27 (1.11–1.46)	1.11 (1.01–1.22)
Low TIBC HR (95% CI)	NA	1.00 (0.77–1.30)	NA	NA
High TIBC HR (95% CI)	NA	1.46 (1.06–2.01)	NA	NA
Low Ferritin HR (95% CI)	1.20 (0.81–1.78)	NA	1.33 (0.98–1.79)	1.10 (0.92–1.31)
High Ferritin HR (95% CI)	1.21 (0.93–1.57)	NA	1.19 (0.92–1.52)	1.05 (0.90–1.52)

Abbreviations. BSI: bloodstream infections, TSAT: transferrin saturation, TIBC: total iron binding capacity.

HR: hazard ratio, CI: confidence interval, OR: odds ratio, NA: not available.

a Adjusted Cox regression analyses comparing five categories of serum iron, TSAT and ferritin levels with the 26th—74th percentile as the reference group.

b Adjusted Cox regression analyses comparing values of serum iron, TSAT and TIBC ≤2.5 percentile or ≥97.5 percentile with the values in between categorised into quintiles. The middle quintiles 40th—60th for all iron status measures were the reference group.

In our study, a higher risk of BSI was observed in those with very low serum iron (≤6 μmol/L), TSAT (≤9%) and high TIBC (≥82 μmol/L) compared to the middle quintile (40th─60th). The HUNT2-study observed a U-shaped risk profile for iron status in the cumulative incidence of BSI, suggesting that both high and low serum iron and TIBC were associated with a higher incidence of BSI. In analyses of BSI-related mortality, low numbers of deaths precluded precise estimates, but indicated that persons with ID had increased risk of dying after an episode of BSI. The results from the two identified population-based studies were confirmed in a systematic review of six small-scaled studies, 51 to 521 hospitalised patients, where ID was associated with higher susceptibility to infections. Four of these studies found either a higher risk of postoperative, respiratory tract, or ICU-acquired infections.^66^ A randomised clinical trial (RCT), including 2157 individuals aged >70 years, found that ID, measured by levels of serum transferrin receptor at enrolment, was associated with 63% higher risk of being hospitalised due to incident severe infection compared with participants without ID (incidence rate ratio of 1.63, CI 1.11–2.41).^67^

Hamilton et al. analysed 72,865 (32.8%) UK Biobank participants with ferritin measurements. Increasing ferritin was associated with higher odds of sepsis (odds ratio (OR) 1.05, CI 1.04–1.06 for every 100 μg/L increase in ferritin). When investigating non-linearity, they showed an increased sepsis risk in both extremes of the ferritin levels. They evaluated sex-stratified analyses and found that women had higher risk of sepsis with the same ferritin level compared to men.^14^ A recent Danish study of blood donors included 94.628 adult participants with ferritin measurements. The study found an increased risk of infections (defined as having antibiotics prescribed) in female iron deficient blood donors (ferritin values < 15 μg/L) (HR 1.08, CI 1.02–1.15), but not for men. For men they identified increased infection risk associated with iron overload (ferritin >300 μg/L) with an HR 1.23 (CI 1.03–1.46). They found no association between iron deficiency and being hospitalised with an infection for both women and men.^68^ Of importance, in Hamilton et al. those with available ferritin test exhibited a different pattern of comorbidities than those without, reflecting that request for ferritin testing was conditional on underlying illness. For both studies, using only high ferritin as an indicator of ID, iron accumulation or increased iron stores could be problematic as ferritin is heavily affected by inflammation.^6^^,^^54^ Even if the regression models were adjusted for potential confounders, the findings in these studies must therefore be read with caution as the observed risk of infections may not be attributed only to iron status.

Mottelson et al. included assessments of risk of infections in participants genotyped for haemochromatosis. They showed that participants homozygous for the *HFE* C282Y variant (n = 422) had increased risk of any infections (HR 1.40, CI 1.16–1.68), and increased risk of sepsis (HR 1.69, CI 1.05–2.73) compared to non-carriers. The *HFE* C282Y variant (refers to the SNP rs1800562 in Table 3) leads to reduced hepcidin production and increased serum iron. Intriguingly, when stratifying on serum iron, TSAT and ferritin levels, the increased risk of any infections was evident even in *HFE* C282Y homozygotes with normal iron status.^11^ A limitation to this finding is the lack of stratification of treatment status in with the potential that normal iron status is transitional and tending to high TSAT during phlebotomy. The finding suggests that low levels of hepcidin, or impaired hepcidin regulation in *HFE* C282Y homozygotes, might impact the homoeostatic control of iron status and iron storage, including handling of iron fluxes during an infectious threat.^11^^,^^26^ Treatment goals for haemochromatosis should include keeping serum iron at stable middle levels, and new therapies to control TSAT in haemochromatosis should be considered.^11^^,^^71^

**Table 3:** Overview of iron related SNPs and the predicted gene included in the MR studies.

SNPs included in MR studies	Gene^a^	Iron status marker	Position (hg38)^b^	SNPs included in MR studies	Gene^a^	Iron status marker	Position (hg38)^b^
The first four SNPs are related to all iron status markers				rs7385804	*TFR2*	Serum iron, TSAT, TIBC	chr7:100638350
rs1799945	*HFE*	Serum iron, TSAT, TIBC, Ferritin	chr6:26090950	rs2529440	*NOD1*^c^	Ferritin	chr7:304722178
rs1800562	*HFE*	Serum iron, TSAT, TIBC, Ferritin	chr6:26092900	rs4841429	*RP1L1*	Ferritin	chr8:10711019
rs57659670	*DUOX2*^c^	Serum iron, TSAT, TIBC, Ferritin	chr15:45106240	rs1495743	*NAT2*	TIBC	chr8:18415780
rs855791	*TMPRSS6*	Serum iron, TSAT, TIBC, Ferritin	chr22:37066796	rs13253974	*SLC25A37*	Ferritin	chr8:23520397
rs75965181	*WNT4*	Ferritin	chr1:22257509	rs7865362	*B4GALT1*	Ferritin	chr9:33117967
rs35945185	*LEPR*^c^	Serum iron, TIBC	chr1:65671556	rs17476364	*HK1*	Ferritin	chr10:69334748
rs469882	*ZNF644*	TIBC	chr1:91064875	rs12419620	*TH/ASCL2*	Ferritin	chr11:2211324
rs10801913	*VANGL1*	Ferritin	chr1:115671658	rs12807014	*FNBP4*	Ferritin	chr11:47738526
rs2228145	*IL6R*^c^	Serum iron, TIBC	chr1:154454494	rs4938939	*MS4A7*^c^	Ferritin	chr11:60393365
rs6025	*F5*	TIBC Ferritin	chr1:169549815	rs174546	*FADS1*	TIBC	chr11:61802356
rs551459670	*IARS2*^c^	Ferritin	chr1:220115348	rs996347	*EGLN3*	Ferritin	chr14:33941688
rs1260326	*GCKR*	Ferritin	chr2:27508076	rs17580	*SERPINA1*	TIBC	chr14:94380927
rs6757653	*WDR43*^c^	Ferritin	chr2:28948938	rs3743171	*SLC24A1*	Ferritin	chr15:65624188
rs12693541	*SLC40A1*	TIBC, Ferritin	chr2:189553962	rs9921222	*AXIN1*	Ferritin	chr16:325782
rs12693541	*SLC40A1*	Ferritin	chr2:189553970	rs3747602	*ZNF500*	Ferritin	chr16:4752382
rs1250259	*FN1*^c^	Ferritin	chr2:215435759	rs535064984	*ASGR2*	Ferritin	chr17:7116978
rs13007705	*ERFE*	Serum iron, TSAT, TIBC	chr2:238160555	rs55789050	*GLPR2*^c^	Ferritin	chr17:9890100
rs34216132	*GNL3*	Ferritin	chr3:52693658	rs34523089	*RNF43*	Ferritin	chr17:58358748
rs7630745	*LRIG1*^c^*/(SLC25A26)*	Serum iron, TIBC	chr3:66376605	rs77262773	*ABCA5*	Serum iron, TIBC	chr17:69253570
rs4854760	*TF*	Serum iron, TSAT, TIBC	chr3:133779797	rs1542752	*OTOP3*	Ferritin	chr17:74941006
rs1131262	*RYK*	Ferritin	chr3:134222476	rs708686	*FUT6*	Ferritin	chr19:5840608
rs3817672	*TFRC*	TSAT	chr3:196073938	rs4808802	*ELL*	Ferritin	chr19:18467063
rs3817672	*TFRC*	TIBC	chr3:196073940	rs2005682	*HAMP*^c^*/FFAR2*	Serum iron, TSAT, TIBC	chr19:35456759
rs59950280	*HGFAC*	TIBC	chr4:3450006	rs601338	*FUT2*^c^	Ferritin	chr19:48703417
rs3743171	*SLC24A1*	Ferritin	chr5:65624180	rs143041401	*CGB5*	Ferritin	chr19:49046858
rs36184164	*VEGFA*	Ferritin	chr6:43813355	rs112727702	*PRPG2*	TIBC	chr19:49587946
rs9399136	*MYB*^c^*/HBS1L*	Serum iron, TSAT, TIBC	chr6:135081200	rs1132274	*RRBP1*	TIBC	chr20:17615508
rs12718598	*IKZF1*	Serum iron, TSAT, TIBC	chr7:50360747	rs6029148	*MAFB*	Ferritin	chr20:40495768

SNPs; Single Nucleotide Polymorphisms.MR; Mendelian Randomisation. TSAT; transferrin saturation. TIBC; total iron binding capacity. chr; chromosome.

a Predicted causal gene identified in Bell et al. and UCSC Genome Browser using GRCh38/hg38.^69^

b Chromosome number: position in GRCh38/hg38.

c High-confidence predicted causal gene based on a variant-to gene algorithm used by Bell et al.^70^

In a subset of 32,141 participants, Mottelson et al. reported on repeated measurements of serum iron and TSAT with a median of 10 years between blood samplings. The median change in serum iron was 3.2 μmol/L (inter quartile range (IQR) 1.7–5.9) and for TSAT 6% (IQR, 3–10%). To our knowledge, this is the only published study that has investigated infection risk in an adult population with repeatedly measured iron status. Higher risk of any infection was identified for participants with low plasma iron and low TSAT in their second blood sample.^11^

To the best of our knowledge, there have been no prospective, population-based studies assessing iron status and risk of COVID-19.

## Iron status as a risk factor for sepsis and COVID-19 in mendelian randomisation studies

Due to the paucity of population-based studies investigating iron status and risk of severe infections, some efforts have been made to use MR studies. Even with meticulous adjustments for confounders, observational association studies remain prone to residual confounding. MR studies serve as an increasingly important tool for studying causal relations in observational epidemiology because MR exploits the principle that genotypes are not susceptible to reverse causation and confounding bias, as they are inherited randomly at conception.^72^ The steps in conducting an MR study and the methods used for MR studies have been explained by Burgess et al. MR uses genetic instruments that are constant throughout to estimate lifetime effects of the exposure,^73^ though the MR methods might not perfectly capture lifetime effect with time-varying exposures.^74^ Three assumptions must be met for an MR study to be valid; the genotype(s) are directly associated with the exposure, the genotype(s) are not related to confounders of the exposure-outcome association, and the genotype(s) affect the outcome only through the risk factor.^14^^,^^73^ Some argue that assessing genetic variants associated with higher iron status could serve as a proxy for an RCT investigating infection risk from iron supplementation.^14^^,^^75^ Another advantage assessing iron status in an MR framework is the potential to investigate long-term effects of genetically predicted iron status on risk of severe infections as severe infections are rare events.

As a basis for performing MR studies on iron status, a large genome wide association study (GWAS) with independent genetic determinants of serum iron, TSAT, TIBC and ferritin using up to 246,293 European subjects, was released in 2021^70^ and Genetics of Iron Status Consortium with 48,927 European subjects, was published in 201 by Benyamin et al.^76^ The SNPs with associated genes related to iron status identified in these GWAS are presented in Table 3. For this review, the SNPs have been revisited using UCSC Genome Browser.^69^ Four MR studies indicate that genetically predicted higher iron status increases the risk of sepsis,^12^, ^13^, ^14^^,^^75^ and two MR studies have identified the same pattern for severe COVID-19.^12^^,^^15^

All MR studies included in this review have stated the aim of the investigation. Only Single Nucleotide Polymorphisms (SNPs) strongly associated with iron indices, sepsis or relevant COVID-19 outcomes were used. The SNPs are checked either by reporting the F statistics (i.e., weak instrument bias), R^2^ (explained variance) and test for independence among SNPs using the linkage disequilibrium panel. To avoid spurious association caused by population stratification both exposure and outcome cohorts must include participants of the same ancestry–all are European. The main MR analysis in all studies was the inverse variance weighted (IVW) which is considered to be the analysis with the greatest statistical power, but dependent on the validity of SNPs and have a balanced pleiotropy.^72^ For the sensitivity analyses, the recommendation is to check for heterogeneity (e.g., Cochran's Q and/or MR PRESSO), perform robust methods (e.g., MR Egger, weighted median, weighted mode, simple mode, and/or MR PRESSO), to check for horizontal pleiotropy (e.g., investigating potential confounding pathways using online tools like GWAS catalogue, Phenoscanner, Ensembl BioMart reported in the included studies) and supplemental analyses like MR PRESSO, MR Egger and multivariable MR. Inclusion of bidirectional MR is a way to assess if sepsis or COVID-19 have causal effects on iron status levels, an indication reverse causation.^72^^,^^73^ The included exposure GWAS and outcome GWAS, with the applied MR analyses is summarised in Supplemental Table S1.

**Fig. 1:**
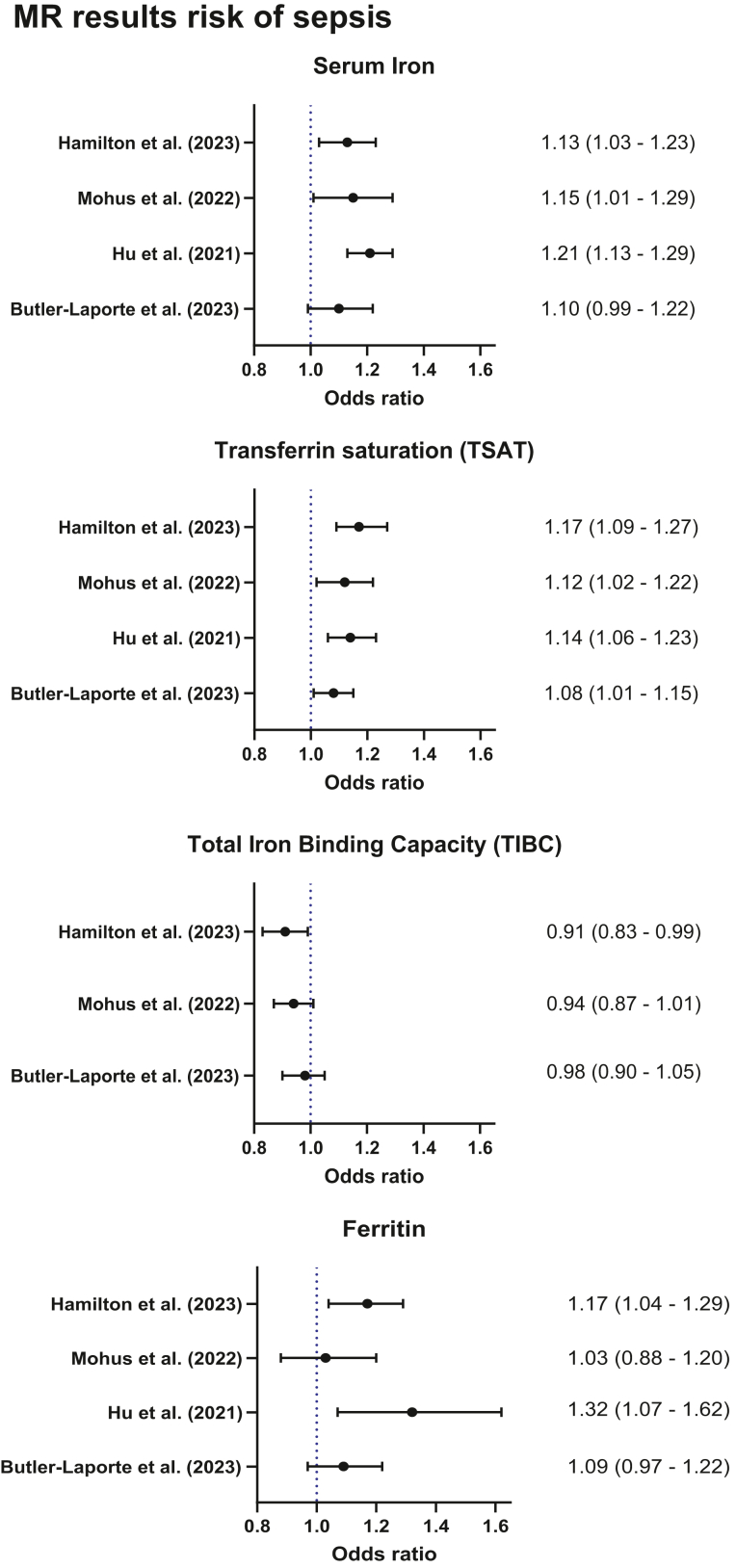
Forest plot of the included MR studies investigating iron status and risk of sepsis with Odds ratios (95% Confidence intervals) obtained from the original studies.^12^, ^13^, ^14^^,^^75^

Previously, we performed a two-sample MR study, where the role of genetically predicted iron status on risk of sepsis and being hospitalised with COVID-19 were assessed using SNPs related to iron status. The results pointed in the direction of a 15% and 12% higher risk of sepsis for each standard deviation (SD) increase in genetically predicted levels of serum iron and TSAT, while lower TIBC was associated with sepsis as expected.^12^ Hu et al. showed that genetically predicted higher levels of serum iron, TSAT, and ferritin were associated with a higher risk of sepsis.^13^ Hamilton et al. found 13% increased risk of sepsis for one SD increase in serum iron, 17% for TSAT and ferritin, again with the expected opposite effect for TIBC.^14^ A forest plot of the MR results related to iron indices and sepsis in the different studies are presented in Fig. 1.

Butler–Laporte et al. performed MR analyses to explore the impact of iron supplementation in the population and risk of infections. Their conclusion was that increasing iron stores would not likely lead to a large increase in the risk of infections. Nevertheless, their MR results point to an increased risk of bacterial infections and sepsis in individuals with genetically predicted higher serum iron and TSAT. Given that they could only identify one genome-wide significant SNP for sepsis, their results must be read with caution.^75^

For COVID-19, we identified two MR studies investigating iron status, both suggesting an effect on COVID-19 outcomes.^12^^,^^15^ Tian et al. found that genetically predicted liver iron status was linked to higher COVID-19 mortality (OR 1.38, CI 1.05–1.82).^15^ In our MR study, each SD increase in serum iron and TSAT showed a 29% higher risk of being hospitalised due to COVID-19 compared to non-hospitalised cases, whereas TIBC and ferritin showed inconclusive results. In sex-stratified analyses, higher serum iron (OR 1.63, CI 0.94–2.86) and TSAT (OR 1.31, CI 0.99–1.75) suggested a more harmful effect in women regarding COVID-19 hospitalisation.^12^

MR studies rely on strong assumptions and there are limitations to MR studies.^73^ Importantly, the included studies have used relevant SNPs for the exposure variants of iron status^70^^,^^76^ or liver iron.^15^ The GWAS on sepsis have used explicit sepsis^12^^,^^13^ or an even more narrow definition on hospital discharge ICD-9/10 codes.^14^^,^^75^ For the two COVID-19 MR studies, different COVID-19 outcomes were used,^12^^,^^15^ meaning that direct comparison of the results is difficult. The U-shaped risk curve might cause an attenuated association when evaluated as linear in two-sample MR analyses, but to date non-linear MR methods are not available.^77^ Iron status fluctuates during a lifetime due to periods of higher demands or losses.^74^ These fluctuations vary according to sex making sex-stratified analyses important. When performing two-sample MR, both the exposure and outcome GWAS must be conducted in populations with similar ancestry to avoid population stratification.^73^ The MR studies have been performed in European populations, and to date no MR studies assessing iron status and risk of severe infection has been conducted in other populations. This is of great concern since ID and severe infections are more prevalent in other populations.^64^

In summary, the included MR studies point in the direction that genetically predicted higher iron status will increase the risk of sepsis, being hospitalised with COVID-19 and COVID-19 mortality. The findings give important information for future RCTs on iron supplementation and fortification programs, but also the urgent need for genetic studies in populations more at risk.

## Interpretation and clinical relevance

A specific challenge when performing epidemiological research, is that only a proportion of exposed individuals develop clinical disease. Even if an individual has the risk factor(s) or the genetic variant(s) being investigated, they must first be exposed to and infected by the pathogen before they could develop the disease being studied.

The population studies tend to show that ID is a risk factor for severe infection,^10^^,^^11^^,^^66^^,^^67^ while the MR studies point to high iron status being a risk factor.^12^, ^13^, ^14^^,^^75^ Importantly, when increasing the numbers of participants under study, as Mottelson et al., they identify a U-shaped risk pattern for the severe infection outcomes, where both extremes of low and high levels of serum iron and TSAT were associated with risk of being hospitalised with any infection.^11^ The same U-shaped risk pattern was observed for BSI when exploring levels of serum iron and TIBC in cumulative incidence analyses in HUNT2.^10^ Hoffman et al. showed a U-shaped correlation between serum iron and bacterial load in a preclinical study on anaemic vs non-anaemic mice comparing Salmonella infection after iron supplementation.^37^

In epidemiological studies the observed associations may be due to variability in the data or random error, and the associations are prone to bias or confounding. Large sample sizes will reduce the risk of random error and potentially generate more precise estimates. Confounding arises from factors that are associated with both the outcome and the exposure, which can bias the true relationship if not properly accounted for. Misclassification of the confounders could lead to underestimation, overestimation or an association pointing in the opposite direction of the true effect.^65^ Meticulous considerations regarding adjustment for confounders are therefore mandatory. The applied Cox regression adjusted for confounders in main analyses with a range of sensitivity analyses and rendered similar estimates.^10^^,^^11^ In Mohus et al. we also omitted cancer related BSI-events, and BSI cases with inflammatory bowel disease and rheumatic diseases in separate sensitivity analyses. To address reverse causation bias from undiagnosed pre-clinical diseases, we performed sensitivity analyses where follow-up started two years after inclusion in HUNT2. In Mottelson et al. the Cox regression analyses were adjusted for levels of CRP, and they performed sensitivity analyses omitting participants with baseline CRP >10 mg/L, anaemia, neutropenia or any comorbidity. All analyses gave similar results to the main analysis. With long follow-up, competing risk by death could become an issue which was assessed using cumulative incidence^10^ and Fine–Grey competing risk regression.^11^

However, residual confounding is always a limitation when interpreting results from population studies and leave arguments for exploring the association in an MR framework. A sub-analysis of anaemic participants (i.e., often related to ID) compared to non-anaemic in Hamilton et al., showed that just small increases in iron status place the individual prone to sepsis.^14^ The observed risk of sepsis could be related to iron metabolism in persons with iron deficiency anaemia (IDA), the cause of IDA (being absolute or functional), and/or the person's handling of iron supplement. Unfortunately, their study did not have information on iron supplementation intake.

Together these findings raise several issues. It could be that long-standing ID puts an individual at risk of infections, further at increased risk of developing BSI, sepsis, or COVID-19 due to suppressed or altered immune functions. Immune defence mechanisms may be relatively more depressed than the ability of bacteria and viruses to sequester iron in low iron environments. This could explain why ID measured prior to a BSI episode or being hospitalised due to any severe infection was a risk factor in the observational studies.^10^^,^^11^^,^^14^^,^^67^ For high iron status, the increased risk of severe infections is likely caused by higher availability of iron for the pathogens which potentially overwhelm the host's immune system,^78^ leading to a rapid growth, colonisation and potentially invasion of pathogens.

For survivors of sepsis and COVID-19, protracted disruptions in iron homoeostasis is related to reduced functional capacity.^16^^,^^25^ It is likely that the profound inflammatory state leave survivors vulnerable to long-term consequences of iron restricted metabolism, including risk of long-term sequela, severe re-infections and death.^57^

An interesting finding in the MR studies is the observation that the higher infection risk starts within the normal range of the iron indices, though at the higher end.^12^^,^^14^ When combining these MR results and higher infection risk in *HFE* C282Y homozygotes with normal iron status, it could indicate that infection risk is influenced by more than just iron levels: the ability to regulate iron fluxes in response to stimuli and how this is executed is also important. For *HFE* C282Y homozygotes, their defective hepcidin regulation could weakened iron-related immune responses.^11^^,^^26^ Another study showing the important role of hepcidin was an RCT of ∼400 rural Gambian infants with 12 consecutive weeks of iron status measurements, investigating the effect of either iron-containing or non-iron-containing micronutrient powders on hepcidin levels. Hepcidin levels were influenced by mild inflammation and were sufficiently raised to impair absorption of iron. Their findings indicate that attempts to combat ID also need efforts to eliminate low-grade inflammation with high hepcidin levels.^53^

Altogether, the included population studies and MR studies place both low and high iron status as a risk factor for severe infections. ID is a global concern, nevertheless the burden of ID is still highest in populations where infection risk is high. The included population studies are from Scandinavia, and the MR studies uses European ancestry only. These epidemiological studies show mounting evidence that ID at the population level needs targeted research, with an emphasis that we need both population-based and genetic studies in the populations most at risk. The findings support the idea that in iron deficient populations, iron supplementation and fortification programs should be accompanied by infection monitoring and control measures.^79^

Iron overload, including persons with mildly raised iron status, persons receiving iron supplements, and persons having or being at risk of haemochromatosis, also show evidence at the population level of increased risk of severe infections. Targeted research is warranted to understand the degree of risk and the mechanistic understanding of how different iron status setpoints, and changes in iron homoeostasis, place persons in risk of infections.

A major challenge when investigating iron status as a risk factor is the profound physiological changes in iron metabolism at the time of infection. Because of this physiological change in iron homoeostasis, we cannot compare the results from population-based studies with studies where iron status was measured at the time of infection. Low iron status measured at hospitalisation could be an appropriate physiological response to the infectious threat. For patients with high iron status at hospitalisation, the studies on iron and risk of sepsis and COVID-19 may indicate that the risk of a severe course or death is due to a more severe inhibition of innate immune functions (as they are not able to withhold iron from invading pathogens), or a relative inability to regulate hepcidin and iron correctly.^18^^,^^20^^,^^59^^,^^60^ Experimental studies show that pathogens can grow more and cause more severe infections under conditions with more iron available.^33^^,^^37^ In this context, the specific type of iron, (i.e., non-transferrin bound iron, or free haem), may be particularly important, as it can be relatively reactive and cause cell and tissue destruction and available for bacterial capture.^78^ The same altered physical conditions may affect the ability of polymorphs to kill bacteria: the phagocytic cells become overwhelmed, and the bacteria outgrow the phagocytic capacity.

Another challenge with the epidemiological studies assessed, is the oversight of fluctuations in iron status during our lifetime, during periods of higher demands, situations with increased losses, or due to chronic medical disorders with immune activation.^6^^,^^35^ Iron status was measured up to 15 years before the outcome. However, any potential misclassification would be non-differential (i.e., not related to later risk of BSI/sepsis, and likely led to an underestimation of the associations).^10^^,^^11^ The MR framework can help overcome limitations from residual confounding and reverse causation and point to genetically predicted higher iron status being related to risk of sepsis and severe COVID-19.^12^, ^13^, ^14^, ^15^^,^^75^

Iron status is truly a double-edged sword, where iron supplementation and treatment with iron chelators must be balanced against harmful effects. Understanding this complexity is crucial for optimising supplementation and fortification strategies, their potential of reducing infectious diseases, but also to target iron status during severe infections. Special attention should be given to individuals during periods of higher demands (i.e., pregnancy), and individuals at risk of ID (i.e., infants in low-income and middle-income countries).^8^

During infections, several treatments have been proposed; iron chelators, ferroptosis antagonists, hepcidin agonists and antagonists, and vaccines targeting bacterial iron acquisition systems.^27^^,^^28^^,^^78^^,^^80^ As iron status changes in the course of severe infections and diurnally, important considerations when performing clinical trials targeting aspects of iron metabolism will be; correct timing of treatments, appropriate concentrations, administration routes (e.g., oral versus intravenous), adverse effects, sex differences, and safety. To date, existing evidence lack specificity about target populations, iron status thresholds and timing of interventions. Unless these factors are understood and considered, it may be difficult to identify individuals who will benefit from iron interventions and equally to characterise situations in which the same interventions might be harmful.

## Outstanding questions

Key questions remain unanswered; Could long-standing ID render individuals more at risk of infection? Which demographics and populations globally are most affected? Could ID during infancy alter haematopoiesis and affect important immune functions with lifelong changes in immunity? Does different iron status setpoints affect risk of infections differently in the sexes? During severe infections, could an individual's ability to cope with the toxicity of free iron due to cell and tissue destruction be affected by the individual's pre-infection iron status?

## Concluding remarks

We have substantial knowledge of iron metabolism and there are evident strong effects of iron on important functions within our immune system. However, iron status at the population level and how this relates to severe infections is not well understood. The lack of population studies with repetitive iron status assessment is limiting our insight into the pathophysiology of iron metabolism making longitudinal analysis of chronic effects of iron status variation difficult. This is of significant concern when iron-status-related disorders rank as one of the leading causes of disadvantage and disability in the world.^9^ Recent population studies indicate that both low and high iron status are associated with significant risk for severe infections in predominantly Caucasian populations. Access to large longitudinal population-based studies, including GWAS consortia, in the populations most at risk for iron status pathologies, will be important to understand how this essential trace element shapes human health. Overall, the aim must be to ascertain whether interventions that correct iron deficiency and iron overload decrease susceptibility to sepsis, along with other health benefits.

## Contributors

RMM contributed with conceptualisation of the project, computed the literature search, the data collection and interpretation including data verification, prepared the figure and tables, and writing of the original draft of the manuscript. LTG contributed with data interpretation, writing, review and editing the manuscript. JKD contributed with writing, review and editing of the manuscript. HD contributed with the conceptualisation of the project, data interpretation, writing, review and editing the manuscript. All authors have read and approved the final version of the manuscript and are responsible for the decision to submit the manuscript.

## Declaration of interests

Nothing to declare.
