# Supplementary material for: Iron—sepsis associations in population health revealed by epidemiology
Source: eBioMedicine. 2025 Sep 15;120:105927. doi: 10.1016/j.ebiom.2025.105927 (PMC12466143; doi:10.1016/j.ebiom.2025.105927)
Supplement: Supplementary Table [file mmc1.docx]

**Supplemental Material**

Iron – sepsis associations in population health revealed by epidemiology

Randi Marie Mohus MD PhD^1,2,3^, orcid : 0000-0002-7625-2664

Lise T. Gustad associate professor^1,4,5^ orcid: 0000-003-2709-3991

Jan Kristian Damås professor^1,6,7^, orcid: 0000-0003-4268-671X

Hal Drakesmith professor^8^, orcid: 0000-0002-8503-6103

1. Central Norway Sepsis Research Centre, Norwegian University of Science and Technology, Trondheim, Norway
2. Clinic of Anesthesia and Intensive Care, St. Olavs hospital, Trondheim, Norway
3. Department for Circulation and Imaging, Norwegian University of Science and Technology, Trondheim, Norway
4. Faculty of Nursing and Health Sciences, Nord University, Levanger, Norway
5. Department of Medicine and Rehabilitation, Nord-Trøndelag Hospital Trust, Levanger, Norway
6. Clinic of Medicine, Department of Infectious Diseases, St. Olavs hospital, Trondheim Norway
7. Department of Molecular and Clinical Medicine, Norwegian University of Science and Technology, Trondheim, Norway
8. MRC Translational Immune Discovery Unit, MRC Weatherall Institute of Molecular Medicine, University of Oxford, John Radcliffe Hospital, Oxford, OX3 9DS, UK

Corresponding author; Randi Marie Mohus; [Randi.m.mohus@ntnu.no](mailto:Randi.m.mohus@ntnu.no)

**Supplemental Table 1: MR studies investigating iron status related to sepsis/COVID-19**

| **Author** | **Iron exposure GWAS** | **Outcome GWAS** | **Main MR analyses** | **Supplemental analyses MR analyses** |
| --- | --- | --- | --- | --- |
| Hamilton et al. 2023^1^ | Bell et al. 2021^2^ | Sepsis  UK Biobank (n=12·664)  Sepsis  FinnGen (n=7·463) | IVW | LD panel (R^2^ < 0·01),  Weighted median,  MR Egger,  weighted mode,  simple mode,  MR PRESSO,  Leave-one-out |
| Mohus et al. 2022^3^ | Bell et al. 2021^2^ | Sepsis  IEU open GWAS (n=10·154) | IVW | F statistics >10,  L panel (R^2^ < 0.01),  Weighted median,  MR Egger,  MR PRESSO,  Leave-one-out,  Phenoscanner,  Bidirectional MR |
| Hu et al. 2021^4^ | Benyamin et al. 2014^5^ | Sepsis  IEU open GWAS  (n=10·154) | IVW | LD panel (R^2^ < 0.005),  Weighted median,  MR Egger,  Leave-one-out,  MR PRESSO,  GWAS catalogue/PubMED,  Bidirectional MR,  Cochran’s Q |
| Butler-Laporte et al. 2023^6^ | Bell et al. 2021^2^  Canadian Longitudinal study on aging^6,7^ | Sepsis  FinnGen (n=7·463)  Sepsis  UK Biobank (n=11·468) | IVW  MR Egger | F statistics > 10,  LD panel (R^2^ < 0·001),  Esembl BioMart,  Bootstraped MR Egger,  Weighted median,  Penalised weighted median  Weighted mode MR,  Simple mode MR,  Cochran’s Q |
| Mohus et al 2022^3^ | Bell et al 2021^2^ | Hospitalised COVID-19 vs non hospitalised COVID-19  Host genetics Initiative (n=4·829)  Sex specific summary data COVID-19  UK Biobank  (female n=1·181, male n=1·703) | IVW | F statistics >10,  LD panel (R^2^ < 0.01),  Weighted median,  MR Egger,  MR PRESSO,  Leave-one-out,  Phenoscanner,  Bidirectional MR |
| Tian et al. 2023^8^ | UK Biobank (liver iron)  Benyamin et al. 2014^5^  (ferritin) | Hospitalised COVID-19 vs non-hospitalised COVID-19  UK Biobank (n=2·884)  COVID mortality  UK Biobank  (n=1·001) | IVW  Weighted median  MR Egger | F statistics > 100,  LD panel (R^2^ < 0·01),  MR Egger intercept,  MR PRESSO,  Cochran’s Q,  Leave-one-out |
| MR: Mendelian randomisation, n=number of sepsis or COVID-19 cases, IVW: inverse variance weighted, LD: linkage disequilibrium | | | | |

# References

1. Hamilton F, Mitchell R, Ahmed H, Ghazal P, Timpson NJ. An observational and Mendelian randomisation study on iron status and sepsis. *Sci Rep* 2023; **13**(1): 2867.

2. Bell S, Rigas AS, Magnusson MK, et al. A genome-wide meta-analysis yields 46 new loci associating with biomarkers of iron homeostasis. *Commun Biol* 2021; **4**(1): 156.

3. Mohus RM, Flatby H, Liyanarachi KV, et al. Iron status and the risk of sepsis and severe COVID-19: a two-sample Mendelian randomization study. *Sci Rep* 2022; **12**(1): 16157.

4. Hu Y, Cheng X, Mao H, Chen X, Cui Y, Qiu Z. Causal Effects of Genetically Predicted Iron Status on Sepsis: A Two-Sample Bidirectional Mendelian Randomization Study. *Front Nutr* 2021; **8**: 747547.

5. Benyamin B, Esko T, Ried JS, et al. Novel loci affecting iron homeostasis and their effects in individuals at risk for hemochromatosis. *Nat Commun* 2014; **5**: 4926.

6. Butler-Laporte G, Farjoun Y, Chen Y, et al. Increasing serum iron levels and their role in the risk of infectious diseases: a Mendelian randomization approach. *Int J Epidemiol* 2023; **52**(4): 1163–74.

7. Raina PS, Wolfson C, Kirkland SA, et al. The Canadian longitudinal study on aging (CLSA). *Can J Aging* 2009; **28**(3): 221–9.

8. Tian H, Kong X, Han F, et al. Liver Iron Overload Drives COVID-19 Mortality: a Two-Sample Mendelian Randomization Study. *Biol Trace Elem Res* 2023.
